# C_60_ Bioconjugation with Proteins: Towards a Palette of Carriers for All pH Ranges

**DOI:** 10.3390/ma11050691

**Published:** 2018-04-27

**Authors:** Matteo Di Giosia, Francesco Valle, Andrea Cantelli, Andrea Bottoni, Francesco Zerbetto, Matteo Calvaresi

**Affiliations:** 1 Dipartimento di Chimica “G. Ciamician”, Università di Bologna, via F. Selmi 2, 40126 Bologna, Italy; a.cantelli@unibo.it (A.C.); andrea.bottoni@unibo.it (A.B.); francesco.zerbetto@unibo.it (F.Z.); 2 Istituto per lo Studio dei Materiali Nanostrutturati (CNR-ISMN), Consiglio Nazionale delle Ricerche, via P. Gobetti 101, 40129 Bologna, Italy; f.valle@ismn.cnr.bo.it

**Keywords:** fullerenes, nanohybrids, nanobiotechnology, bioconjugation, chemical stability

## Abstract

The high hydrophobicity of fullerenes and the resulting formation of aggregates in aqueous solutions hamper the possibility of their exploitation in many technological applications. Noncovalent bioconjugation of fullerenes with proteins is an emerging approach for their dispersion in aqueous media. Contrary to covalent functionalization, bioconjugation preserves the physicochemical properties of the carbon nanostructure. The unique photophysical and photochemical properties of fullerenes are then fully accessible for applications in nanomedicine, sensoristic, biocatalysis and materials science fields. However, proteins are not universal carriers. Their stability depends on the biological conditions for which they have evolved. Here we present two model systems based on pepsin and trypsin. These proteins have opposite net charge at physiological pH. They recognize and disperse C_60_ in water. UV-Vis spectroscopy, zeta-potential and atomic force microscopy analysis demonstrates that the hybrids are well dispersed and stable in a wide range of pH’s and ionic strengths. A previously validated modelling approach identifies the protein-binding pocket involved in the interaction with C_60_. Computational predictions, combined with experimental investigations, provide powerful tools to design tailor-made C_60_@proteins bioconjugates for specific applications.

## 1. Introduction

C_60_, the most representative member of the fullerenes family, has steadily attracted interest for its possible use in various fields, including nanomedicine [1,2,3,4,5,6,7]. A plethora of fullerene-based compounds have been synthesized with different targets. They display a range of biological activities that are potentially useful in anticancer therapy, antimicrobial therapy, enzyme inhibition, controlled drug delivery, and contrast or radioactivity-based diagnostic imaging [7,8,9,10,11,12,13]. Noteworthy is the possibility of their use in photodynamic and photothermal therapies [8,14,15]. The photophysical and electrochemical properties of C_60_ depend on their dispersion and a strict control of their disaggregation is truly necessary for nanotechnological applications [16,17,18]. To date two main approaches have been followed to tackle fullerene insolubility in water:

(i) the covalent approach is the most used method to prevent fullerene aggregation. The benefits obtained by functionalization are often offset by reduced photophysical performances [19];
(ii) the noncovalent approach requires the use of supramolecular hosts that are amphipathic molecules able to interact with a single fullerene and to screen it from the aqueous environment. A variety of hosts is capable of interacting with fullerenes. They include surfactants, synthetic polymers, biopolymers, cyclodextrin [20], to name a few. In all cases, they stabilize small clusters of fullerenes [21]. In recent years, also proteins have become used as dispersing agents of fullerenes [9,22,23,24], CNTs [25,26,27,28,29] and graphene [30]. Proteins are naturally amphiphilic. This feature may avoid complicated synthetic procedures or the use of organic solvents. Most proteins are also pH responsive, which is an advantage for some manipulations [26]. Steric hindrance and electrostatic repulsion are the key factors determining the stability of the dispersion of carbon nanomaterials-protein complexes in aqueous solutions [31].

Protein corona [32,33,34] determines the biological fate of ultra-small NPs and nanoclusters and controls physiological responses. From the biological point of view, encapsulation of fullerenes by proteins may control and possibly decrease the cytotoxicity. Well-dispersed fullerenes [35] and CNTs are less toxic than their agglomerates [36]. Protein binding can also alter the cellular pathways of interaction with carbon nanomaterials. Ultimately, coating of carbon nanomaterials with proteins can confer them a new biological identity [37]. 

We recently proposed the use of lysozyme to disperse with a 1:1 stoichiometry C_60_ in water [23]. The hybrid is well-defined and the fullerene binds selectively in the protein-substrate binding pocket. The protein-based supramolecular adduct preserves the photophysical properties of C_60_ and allows the exploitation of C_60_ as a photosensitizer for photodynamic treatments [35]. 

In this work, we show that the non-covalent bioconjugation of C_60_ with proteins offers a palette of carriers for fullerenes for all pH ranges. We evaluate the stability of C_60_@protein complexes in biologically relevant conditions. Two proteins characterized by opposite net charges in physiological conditions are used as model systems and the role of the electrostatic contribution to the stability of their adducts with C_60_ is identified. Applications of docking protocols and Molecular Mechanics Poisson–Boltzmann Surface Area (MMPBSA) calculations [38,39] further provide accurate description of the C_60_ binding pocket involved in the interaction between protein and C_60_.

## 2. Materials and Methods

Trypsin from porcine pancreas (Cat. no. T0303), pepsin from porcine gastric mucosa (Cat. no. P7012), fullerene C_60_ (Cat. no. 483036) were purchased from Sigma Aldrich. They were used without further purifications. Phosphate-buffered saline (PBS) solutions were prepared dissolving the tablets purchased from Sigma Aldrich (Cat. no. P4417) in milli-Q ultrapure water. 

### 2.1. C_60_@Protein Synthesis 

The C_60_@protein hybrids were prepared mixing an excess of fullerene powder with a 0.3 mM solution of each protein (5 mL), with a 2:1 stoichiometry. NaOH and HCl 1M were used to adjust pH of the protein solutions. The heterogeneous mixtures were then sonicated in a vial for 120 min using a probe tip ultrasonicator (Ultrasonic Processor UP200St, Hielscher Ultrasonics GmbH, Teltow, Germany), equipped with a sonotrode S26d7, used at 40% of the maximum amplitude). During the process, the sample was refrigerated with an ice bath. The dark brown turbid mixture obtained after the sonication was centrifuged at 10 kRCF. The resulting supernatant was then collected and characterized. 

### 2.2. C_60_@Protein Characterization

UV-Vis absorption spectra were recorded at 25 °C by means of Cary 60 UV-Vis Spectrophotometer (Agilent, Santa Clara, CA, USA). Surface charge analysis of the hybrids were estimated measuring the zeta-potential at 25 °C by means of Malvern Nano ZS.

Atomic Force Microscopy (AFM) experiments were performed at the SPM@ISMN microscopy facility in Bologna. AFM analysis were done with a Multimode VIII equipped with a Nanoscope V controller (Bruker Nano Surface, Santa Barbara, CA, USA) operated in ScanAsyst mode to evaluate the quality of the monodispersion of the bioconjugates. The samples were prepared by drop casting 10 μL of C_60_@protein solution onto a freshly cleaved mica substrate for 10 min then rinsed with milli-Q ultrapure water and dried under a nitrogen stream.

### 2.3. Computational Protocol 

**Generation of the poses.** Docking models were obtained using the PatchDock algorithm [40]. PatchDock takes as input two molecules and computes three-dimensional transformations of one of the molecules with respect to the other with the aim of maximizing surface shape complementarity, while minimizing the number of steric clashes.

**Scoring of the poses.** Accurate rescoring of the complexes is then carried out using FireDock program [41]. This method simultaneously targets the problem of flexibility and scoring of solutions produced by fast rigid-body docking algorithms. Sidechain flexibility is modeled by rotamers and Monte Carlo minimization [42]. Following the rearrangement of the side-chains, the relative position of the docking partners is refined by Monte Carlo minimization of the binding score function. Free energy of solvation/desolvation in the binding process is taken into account by a solvation model that uses estimated effective atomic contact energies (ACE) [43]. All the candidates are ranked by a binding score [43]. This score includes, in addition to atomic contact energy used to estimate the desolvation energies [43], van der Waals interactions, partial electrostatics, explicit hydrogen and disulfide bonds contribution. In addition, three components to the total binding score are added:E_π-π_ for the calculation of the π-π interactions, E_cation-π_ for the calculation of the cation-π interactions and E_aliph_ for the calculation of hydrophobic interactions.

**Minimizing the pose.** The best poses for every selected protein were full minimized by AMBER 12 [44]. The ff12SB force field [44] was used to model the proteins, while the fullerene atoms were modeled as uncharged Lennard-Jones particles by using the CA atom type (sp^2^ Aromatic Carbon parameter), also from the AMBER force field. The minimization was carried out with sander, using the GB (Generalized Born) model [45] for the solvation and no cut-off for van der Waals and electrostatic was used.

**MM-GBSA analysis.** In order to identify the residues responsible for the binding of the proteins to C_60_, we carried out a decomposition analysis of the optimized structure according to the MM-GBSA scheme [38,39]. The per-residue decomposition analysis provides the contribution of the individual amino acids to the binding.

## 3. Results and Discussions

The ability of C_60_ to interact with proteins is a recent subject of investigation. Collectively, van der Waals, hydrophobic and electrostatic interactions must cooperate to establish energetically favorable interactions between a protein and a fullerene in order to allow the formation of a stable complex [46]. Geometrical complementarity also plays a primary role to maximize the effect of the stabilizing contributes [47]. Crucial for the understanding of protein-fullerene interactions is the identification of the fullerene-binding site together with the possible subsequent proteins structural modification [48]. It should also be further assessed if the interaction occurs between a single fullerene with a single protein or if fullerenes clusters are surrounded by a number of proteins.

Pepsin (pI = 2.2–3) [49] and trypsin (pI = 10.2–10.8) [50] are proteins characterized by very different values of isoelectric point, which makes one negatively and the other positively charged in physiological conditions. Sonication of C_60_ with each protein was performed in acidic (pH 2), neutral, and basic pH (pH 12) of unbuffered aqueous solutions, to avoid stabilizing/destabilizing effects due to the different buffer composition. Pepsin was able to disperse fullerene in water only at basic pH, where the protein is negatively charged, while trypsin showed the best performances at acidic pH.

**Figure 1:**
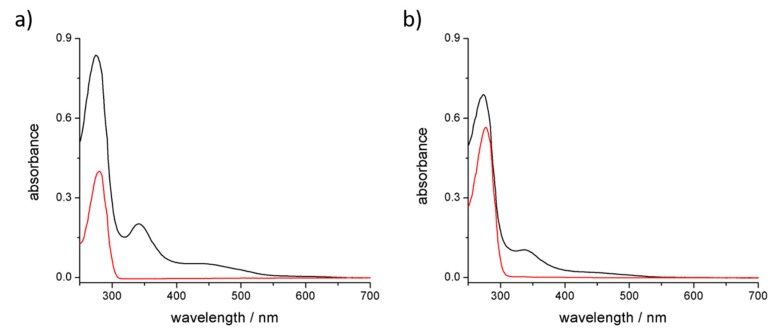
UV-visible spectra of (**a**) C_60_@trypsin (black line) and trypsin (red line); (**b**) C_60_@pepsin (black line) and pepsin (red line).

The two batches of hybrids were synthetized under optimized conditions. After sonication and centrifugation, the supernatants were collected and characterized. UV-Vis spectra of the solutions (Figure 1) show the diagnostic absorption bands of C_60_ at 341 nm and the overlap of C_60_ and protein absorption bands between 260 and 290 nm. Based on the extinction coefficients of both components of the adducts [51], the absorption spectra suggest a 1:1 stoichiometry between C_60_ and trypsin, while 1:2 stoichiometry can be estimated for the C_60_ and pepsin complex. UV-Vis spectra also suggest that the presence of particle aggregates, observed prior to centrifugation, was completely removed since scattering is not exhibited.

### 3.1. C_60_@Pepsin—C_60_@trypsin, an Atomistic View

**Figure 2:**
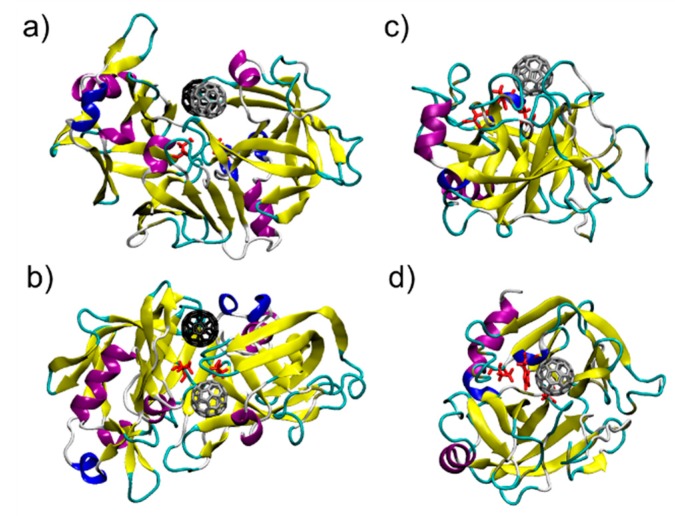
Two perspectives of C_60_@pepsin (**a**,**b**) grey coloured C_60_ occupies the binding pocket 1, black coloured C_60_ occupies the binding pocket 2 and C_60_@trypsin (**c**,**d**). In red, the catalytic residues of the two proteins.

Surface complementarity between the proteins and the C_60_ surface appears. The results of the docking protocol explain the stoichiometry observed by the UV-visible spectra. Pepsin is characterized by a dimeric interface region. In this region, two fullerene binding pockets are identifiable (Figure 2a,b).

**Figure 3:**
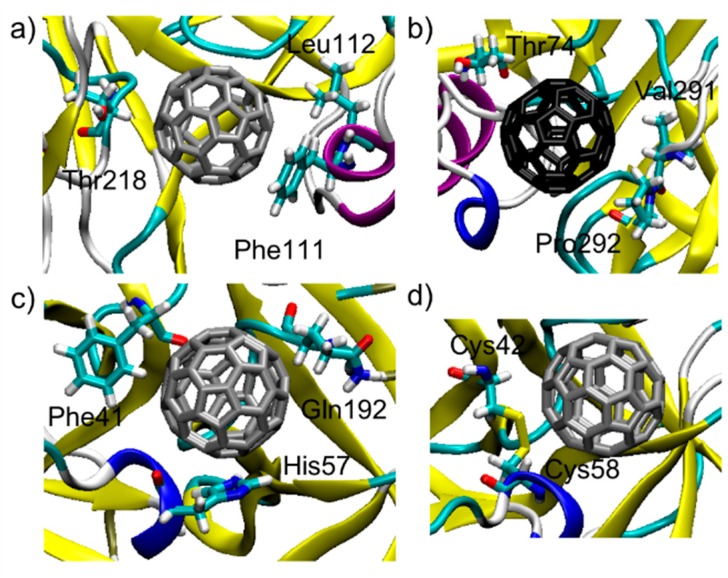
Top 3 residues interacting with C_60_ in the (**a**) pepsin binding pocket 1; (**b**) pepsin binding pocket 2; (**c**) Top 3 residues interacting with the C_60_ in the trypsin binding pocket; (**d**) Interaction in the trypsin binding pocket between C_60_ and a disulfide bridge (Cys42-Cys58).

The binding between C_60_ and pepsin is not surprising, since pepsin is an aspartic protease and structurally strongly correlates to HIV protease: fullerenes are well known inhibitors of HIV-1 protease [52,53,54,55]. In pepsin, as in the HIV protease, fullerenes block the large active site groove [52,53,54,55]. C_60_ is also a known serine protease inhibitor [56], and in fact C_60_ binds in the trypsin active site: a single, well defined binding pocket is identified by the docking protocol in this region (Figure 2c,d). For the two C_60_@protein hybrids tested here, MM-GBSA analysis of the structures in their optimized geometries provides a quantitative description of the C_60_ binding pocket and identifies the more effectively interacting residues. Table 1 shows the 10 largest interactions between the residues of the proteins and C_60_. The three most interacting residue for binding pocket are represented in Figure 3a–c.

From Table 1, Figure 3, and Figures S1–S3 it appears that proteins are able to interact with C_60_ via:

(i) π-π stacking interactions that are established between aromatic residues (phenylalanine, tyrosine, histidine) and C_60_ surface [25,57];
(ii) Hydrophobic interactions (leucine, isoleucine, methionine, proline, glycine) that are established in water between aliphatic residues and C_60_ surface [25];
(iii) Surfactant-like interactions where amphiphilic residues (threonine, serine, aspartate) behave similarly to surfactants and solvate C_60_. The hydrophobic aliphatic chains of these residues interact with C_60_ surface, whereas the hydrophilic groups point out toward water [25,58,59].

In the case of trypsin, of interest is the interaction between a disulfide bridge (Cys42-Cys58) and C_60_ (Figure 3d). This kind of interaction was recently highlight by Hirano and coworkers for carbon nanotubes [60,61].

### 3.2. AFM Analysis of C_60_@Protein Hybrids

UV-Vis spectra and molecular modelling exhibit the expected stoichiometry between C_60_ and proteins. They do not give information about the possible aggregation of the adducts. Atomic force microscopy is a direct technique to evaluate the size distribution of particles.

**Figure 4:**
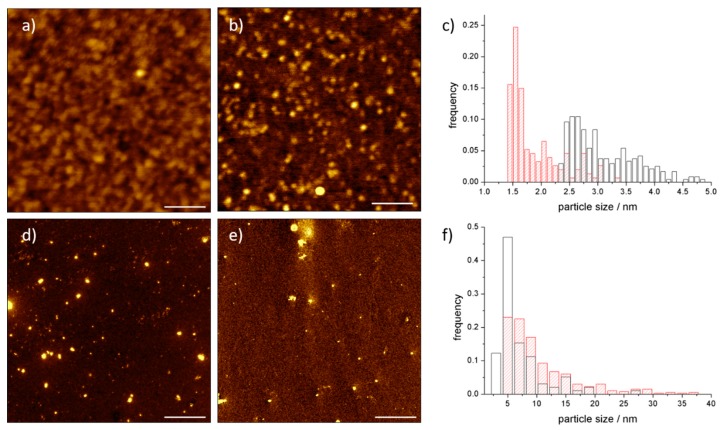
AFM images of (**a**) C_60_@trypsin; (**b**) trypsin and (**c**) height distribution of C_60_@trypsin (red) and trypsin (black). AFM images of (**d**) C_60_@pepsin, (**e**) pepsin and (**f**) height distribution of C_60_@pepsin (red) and pepsin (black). Scale bar (**a,b**) 100 nm; (**d,e**) 1 µm.

In Figure 4a, the C_60_@trypsin hybrids are monomoleculary dispersed when deposited on a negatively charged mica surface. C_60_@trypsin is positively charged; hence, an electrostatic interaction takes place with the surface. The height distribution (Figure 4c) of both C_60_@trypsin and the trypsin reference (obtained in the same conditions) shows an average height which is slightly lower than the expected value. This behavior is a consequence of the strong electrostatic interaction, which squashes the proteins over the surface in order to maximize the attractive electrostatic contacts. Conversely, negatively charged pepsin hybrid (Figure 4f) shows an average height, which is slightly higher than the average size of the protein. These results mainly originate from the combination of different forces: (i) the pepsin tendency to self-associate; (ii) the electrostatic repulsion between the pepsin and the surface, which reduces the number of interactions, as confirmed also by the small number of the particles deposited on the mica which repels the adduct. 

The AFM analysis demonstrates the absence of C_60_@proteins aggregates, or nC_60_ clusters dispersed by the proteins.

### 3.3. Stability of the Complex in Aqueous Media

**Figure 5:**
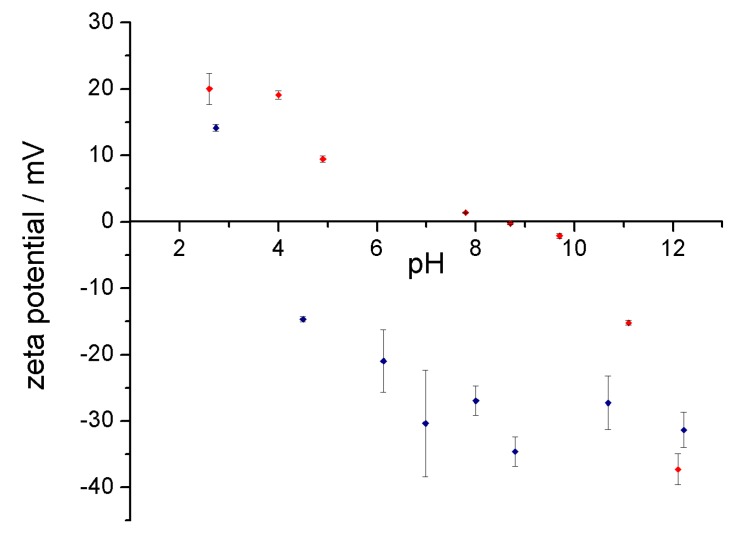
Zeta potential of C_60_@trypsin (in red) and C_60_@pepsin (in blue) hybrids as a function of the pH in aqueous solution. Standard deviations are shown in the error bars.

Compared to the chemical functionalization of the fullerenes, one of the advantages from the use of host-guest system is the possibility to tune the stability of the complex in aqueous media. The tuning can be achieved by acting only on the host system, which is the protein. Evaluating the behavior of C_60_@proteins at different pH’s and physiological conditions, it was found that the stability of the hybrid in aqueous media was completely governed by the protein. To understand if proteins pH sensitivity was retained, acid-basic titration was performed. Zeta potential and UV-Vis spectra were obtained. The correlation between zeta potential and pH gives information about the behavior of the complex for possible future in vivo experiments, since pH varies in different compartments of the organisms. Moreover, the greater the range of pH stability the wider the conditions for subsequent manipulation of the adduct. pH dependent zeta potential trends of C_60_@trypsin and C_60_@pepsin are shown in Figure 5.

The isoelectric points (IEP) of both adducts resulted slightly shifted to values of pH’s closer to neutrality with respect to IEP of the pristine proteins. This phenomenon can be attributed to a reduced accessibility to pH sensitive groups upon fullerene complexation. A further effect is related to the local change of the environment polarity, which could slightly perturb the pKa of few charged residues. Electrostatic repulsion and steric hindrance are two of the major mechanisms active in the dispersion of fullerene in protein solutions. At pH values different from the isoelectric point (IEP), the charge distribution on the atoms of the proteins makes the protein-stabilized C_60_ repel each other. For pH values closer to the IEP, the electrostatic repulsion between the proteins/adducts becomes minimal. The stability of possible aggregates is governed only by steric hindrance.

**Figure 6:**
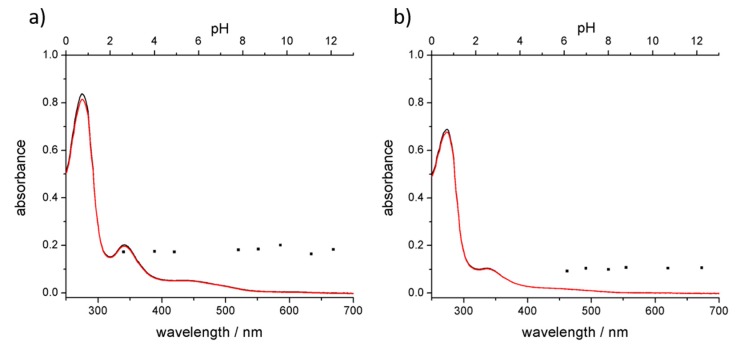
UV-Vis absorption spectra of (**a**) C_60_@trypsin and (**b**) C_60_@pepsin in water (black lines) and PBS (red lines). Black dots represent the absorbance of fullerene diagnostic band (341 nm) of the hybrids at different pH values (top axis).

For C_60_@pepsin complexes at pH values close to IEP (2.7 and 4.5), aggregation phenomena occurred after few minutes. C_60_@trypsin complexes did not aggregate also for pH values close to the IEP. Around the IEP, for C_60_@trypsin, protein steric repulsion provides a barrier to prevent fullerene aggregation. The maximum stability for individual C_60_@pepsin complexes was obtained in neutral and basic conditions. Absorption spectra performed on the same samples did not show changes of shape and intensity (Figure 6) between the different samples. After three months, C_60_@pepsin is perfectly stable while for C_60_@trypsin, more than 90% of the initial dispersed fullerene is detected (Figure S4). 

Comparison of the absorption spectra of C_60_@trypsin (Figure 6a) and C_60_@pepsin (Figure 6b) in water and phosphate-buffered saline solution shows that the hybrids are stable also in physiologically relevant conditions (represented by PBS). This is an important difference with other C_60_ adducts, for instance fullerenes dispersed by cyclodextrins rapidly precipitates when NaCl is added [62]. On the opposite, proteins are stable in a “salty environment” that represents their physiological condition.

These results suggest that fine-tuning of the net charge of the complex is possible and therefore it should also be possible to take advantage of the nature of each protein to create optimal C_60_-protein systems as a function of the pH. Tuning the net charge of the protein used to host the C_60_ molecule it is possible to governs its interactions with cellular and bacterial surface, controlling C_60_ toxicity [63,64,65,66].

## Figures and Tables

**Table 1:** Interaction energies (kcal mol^−1^) of the top 10 residues interacting with C_60_.

C_60_@protein Complex	Top 10 Residues Interacting with C_60_				
C_60_@Pepsin-Binding pocket 1	Phe 111 = −5.7	Leu 112 = −3.1	Thr 218 = −3.0	Ser 219 = −2.9	Thr 12 = −2.8
	Glu 13 = −2.8	Phe 117 = −2.6	Ile 30 = −2.5	Tyr 75 = −2.5	Thr 77 = −2.2
C_60_@Pepsin-Binding pocket 2	Val 291 = −4.9	Thr 74 = −4.3	Pro 292 = −3.7	Tyr 75 = −3.4	Gly 76 = −2.7
	Met 289 = −1.4	Thr 293 = −1.3	Tyr 189 = −1.2	Asp 290 = −1.0	Leu 298 = −0.6
C_60_@Trypsin	His 57 = −4.9	Phe 41 = −4.2	Gln 192 = −3.5	Cys 58 = −3.4	Cys 42 = −2.7
	Gly 193 = −1.8	Ser 195 = −1.7	Asp 194 = −0.8	Tyr 151 = −0.6	Leu 99 = −0.4

## Supplementary Materials

Supplementary materials can be found at http://www.mdpi.com/1996-1944/11/5/691/s1.
