# Supplementary material for: C60 Bioconjugation with Proteins: Towards a Palette of Carriers for All pH Ranges
Source: Materials (Basel). 2018 Apr 27;11(5):691. doi: 10.3390/ma11050691 (PMC5978068; doi:10.3390/ma11050691)
Supplement: Supplementary file 1 [file materials-11-00691-s001.pdf]

C_{60} bioconjugation with proteins: towards a palette of carriers for all pH ranges

Matteo Di Giosia* [1], Francesco Valle [2], Andrea Cantelli [1], Andrea Bottoni [1], Francesco Zerbetto [1] and Matteo Calvaresi [1,*]

[1] Dipartimento di Chimica "G. Ciamician", Università di Bologna, V. F. Selmi 2, 40126 Bologna, Italy;

[2] Istituto per lo Studio dei Materiali Nanostrutturati (CNR-ISMN), Consiglio Nazionale delle Ricerche, via P. Gobetti 101, 40129 Bologna, Italy; f.valle@ismn.cnr.bo.it

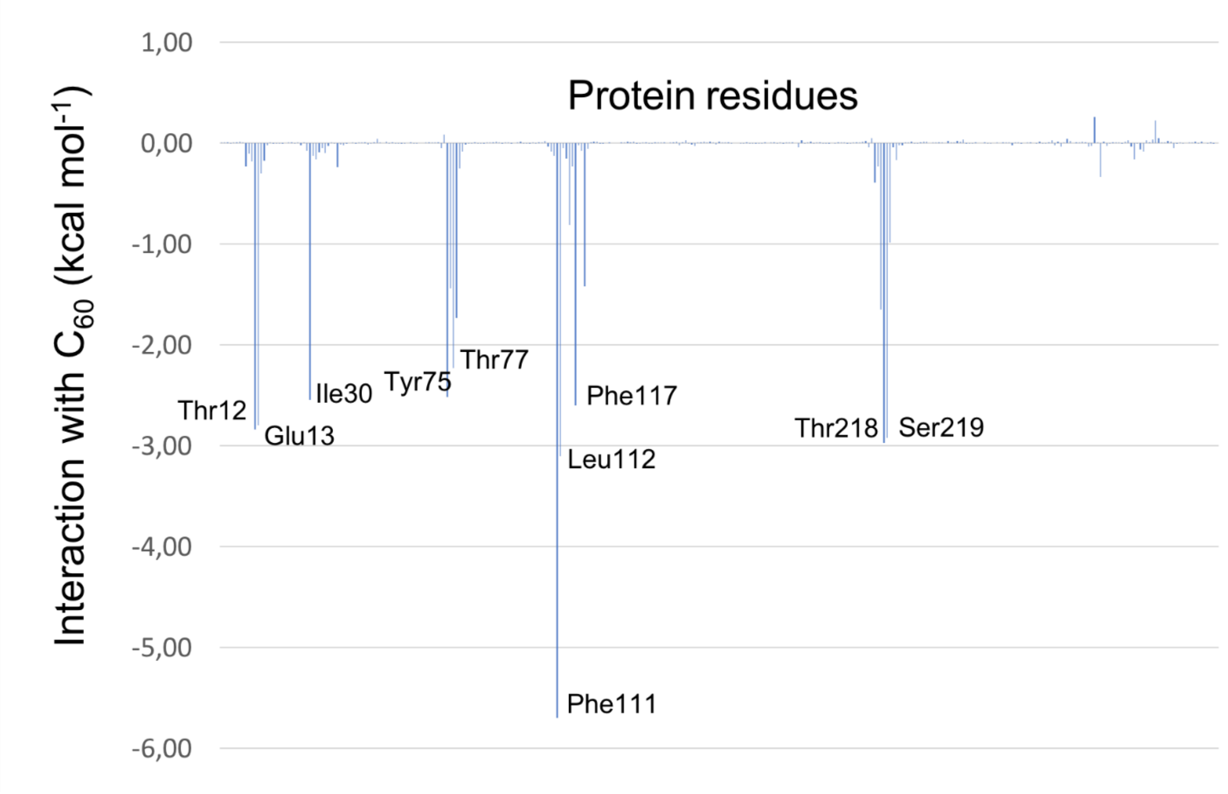

Figure S1. Pepsin binding pocket 1 – C_{60} interactions. $\Delta G_{binding}$ decomposed per residue.

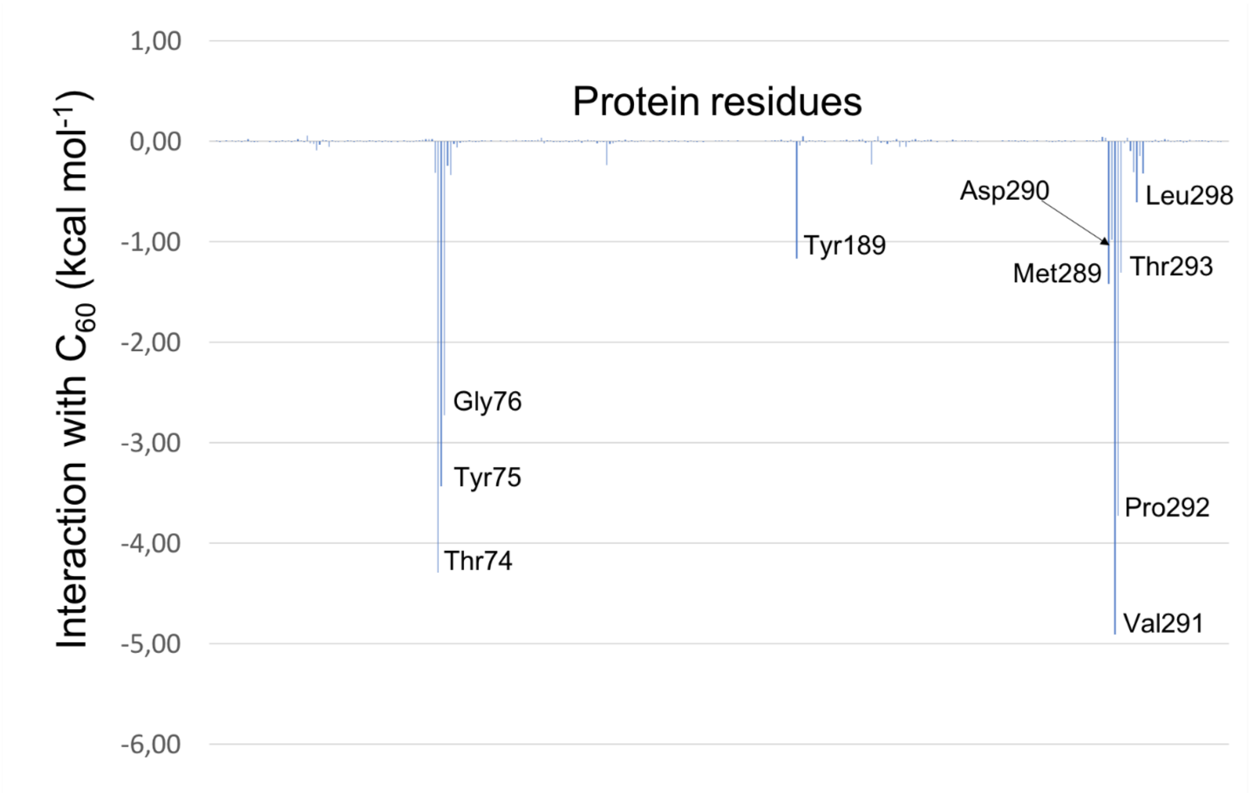

Figure S2. Pepsin binding pocket 2 – C_{60} interactions. $\Delta G_{binding}$ decomposed per residue.

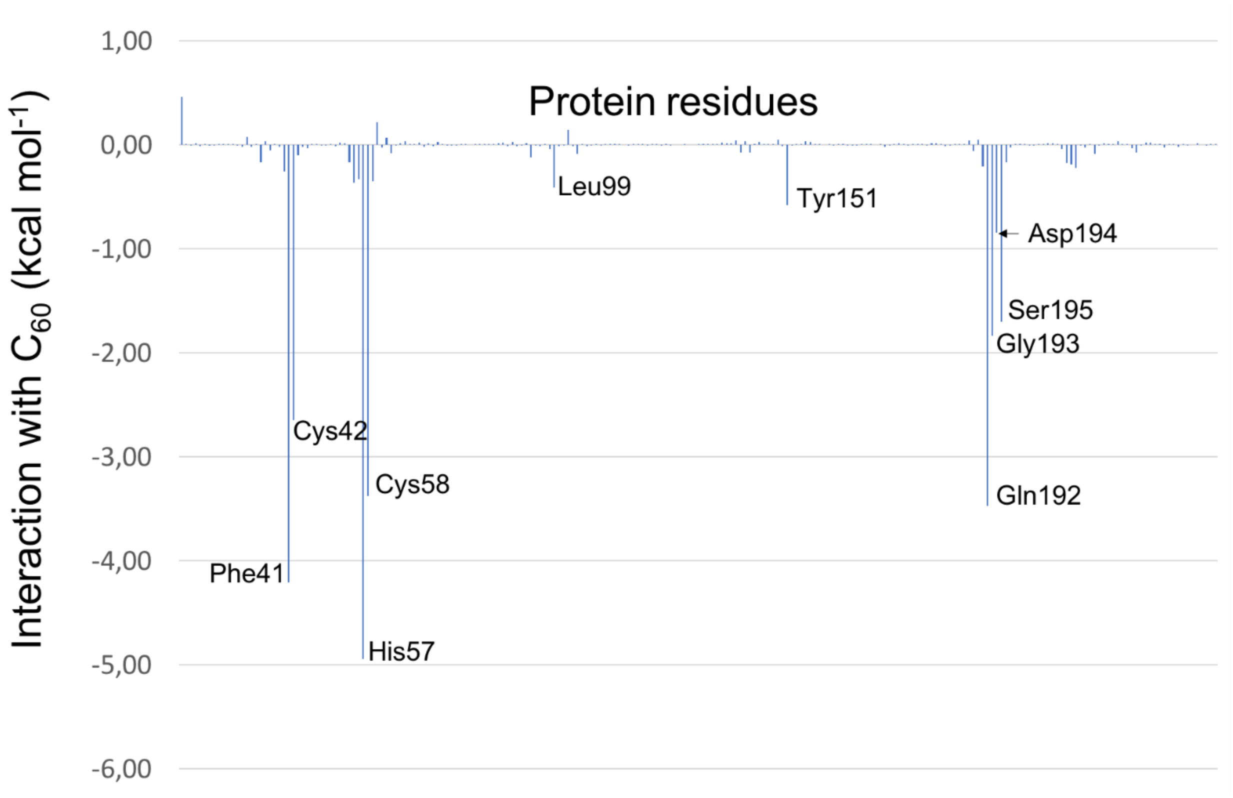

Figure S3. Trypsin – C_{60} interactions. $\Delta G_{binding}$ decomposed per residue.

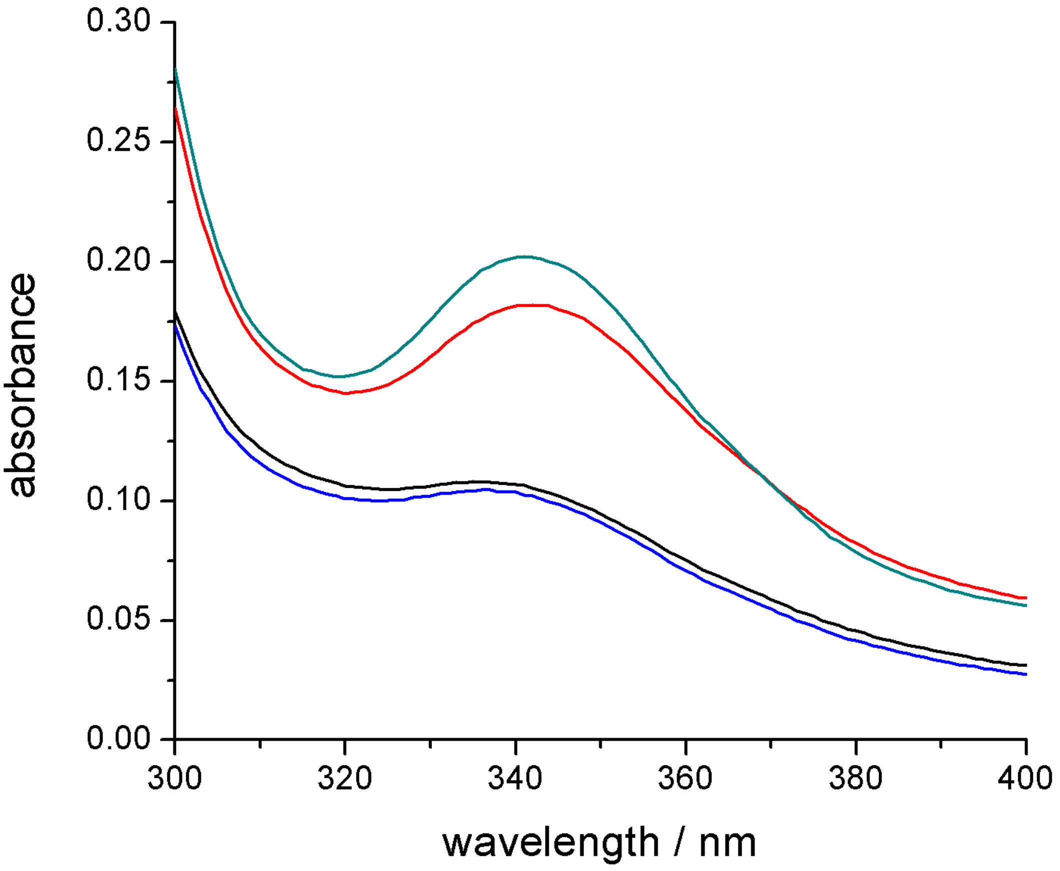

Figure S4. UV-visible spectra of C_{60}@pepsin (after synthesis - black line; after 3 months – blue line) and C_{60}@trypsin (after synthesis - green line; after 3 months – red line).
